# Global research status and trends of UKA for knee osteoarthritis: a bibliometric analysis

**DOI:** 10.1186/s42836-020-00039-3

**Published:** 2020-07-24

**Authors:** Peidong Liu, Chen Zhang, Zhan Lu, Jiangfeng Feng, Wenjie Xu, Ziquan Yang

**Affiliations:** 1 Department of Orthopaedics, Shanxi Medical University Second Affiliated Hospital, Taiyuan, 030000 Shanxi China; 2 Provincial Key Laboratory of Bone and Soft Tissue Damage Repair, Shanxi Medical University Second Affiliated Hospital, Taiyuan, 030000 Shanxi China; 3 Wuxi the Ninth People’s Hospital Affiliated to Soochow University, Wuxi, 355200 Jiangsu China

**Keywords:** UKA, Osteoarthritis, Joint replacement, Bibliometrics, Research trends

## Abstract

**Objective:**

As an alternative of knee-protection surgery, unicompartmental knee arthroplasty has been widely used for the treatment of knee osteoarthritis and has achieved good clinical results. However, reports on its data and trend are scanty. This article reviewed current status and trend in the research of UKA, and compared different regions, organizations and authors in terms of their contributions to the field.

**Methods:**

The literature on UKA ranging from 2009 to 2019 was searched in the “Web of Science” database, and the search results were visually presented by using Excel and VOS-viewer software packages, and the *status quo* and development trends of relevant studies were analyzed.

**Results:**

A total of 1264 articles on UKA were identified, of which 330 were the larger studies conducted in the United States. The institution that published most papers was Oxford University, with a total of 109 papers published. MURRAY DW was the largest contributor in this field. The National Institutes of Health was the largest funding agencies of the UKA. Studies could be divided into six clusters in terms of prosthesis design, follow-up investigation, OA etiology, hip-knee association, joint replacement registration, and computer navigation. “Computer-aided navigation” and “gait analysis” promise to be future hot spots in the field of UKA research.

**Conclusion:**

Global trend analysis suggests that UKA research is gradually deepening and the number of papers has been on the rise. The USA was the largest contributor to this field. More research effort should be directed to “Computer-aided navigation”and “gait analysis”, which might be the popular topics in the UKA field in not very distant future.

## Background

Knee osteoarthritis (KOA) is a degenerative joint disease that is prevalent in middle-aged and elderly people. Its main manifestations are knee cartilage degeneration, osteophyte hyperplasia, peri-knee pain and joint dysfunction [1]. In recent years, with the stepwise treatment of KOA, the standardized preaching and promotion of knee-sparing surgery has popularized the concept of knee-sparing [2]. Unicompartmental knee arthroplasty (UKA), as a technique of knee-sparing surgery [3], has been widely used for the treatment of knee osteoarthritis because of its unique advantages of retaining cruciate ligaments and maintaining the proprioception of the knee, with good clinical results achieved [4, 5].

Bibliometrics is a method that uses literature data as a research object to conduct qualitative and quantitative research on published literature through keywords, titles, authors, institutions, *etc*., to quickly assess the research status of a certain field and predict its development trend [6]. Bibliometric analysis is also gradually being used to develop clinical practice guidelines and analyze the trends in the research of related diseases [7]. UKA has the advantages of less trauma, less blood loss and faster recovery. At the same time, it is conducive to retaining the normal biomechanics of the knee joint and conserving more functionally important structures than TKA. UKA has been extensively employed for the treatment of KOA [8]. However, there is a paucity in the statistics and trend research of UKA in literature.

The purpose of this article was to investigate the trend of UKA research, compare the research contributions of different regions, organizations and authors, provide a statistical reference for related literature research, and conduct a summary analysis to better understand the global UKA research trends and find hot topics in the field to provide informational basis and empirical references for related research.

### Citation data collection

**Fig. 1:**
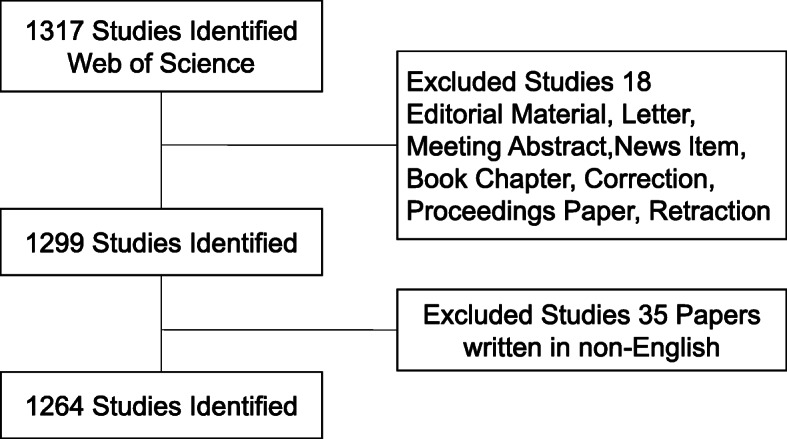
Flow diagram of UKA research selection

The “Web of Science” (WOS) database is considered to be the best database for bibliometric analysis due to its detailed and complete coverage of publication data [9]. This study used the WOS database to search for articles published in the years 2009–2019 by employing the subject term “UKA”. All searches were completed within one day in 2020-03-11 to avoid changes in publication and citations as much as possible. After two authors independently selected all the relevant data for data extraction, the final literature was included in the study. The measurement indicators of data extraction mainly included authors, keywords, nationalities, research institutions, citation frequency, *etc*. [10]. The search expression was as follows: Subject: (“unicompartmental knee arthroplasty” OR “UKA” OR “medial unicompartmental replacement” OR “MUR” OR “lateral unicompartmental replacement” OR “LUR”) NOT subject: (“TKA” OR “total knee arthroplasty”) Fig. 1 Details of the entire retrieval process.

### Bibliometric analysis

Upon analysis of the search results in the WOS database, the publication year, country, source publication, institution, and authors were identified one by one, and results were inputted to Excel for statistical analysis. The relevant data were processed using the drawing function of Excel.

### Data visualization analysis

VOS-viewer software was used to visualize the literature data [11]. Through the “Document Coupling Analysis” option in the software, author coupling analysis, mechanism coupling analysis, and country coupling analysis were performed separately, and the analysis chart was derived. The size of the circle reflects the degree of connection between the literatures [12]. Coupling analysis was used to study the cooperation between institutions and authors, and to indirectly judge its influence on international cooperation [13]. Co-occurrence visualization analysis helps review the related development process of the subject, identify research trends and current hotspots, and therefore plays a very important role in capturing current status of research frontiers [14].

## Results

### Bibliometric analysis of UKA research

#### Global volume forecast model

**Fig. 2:**
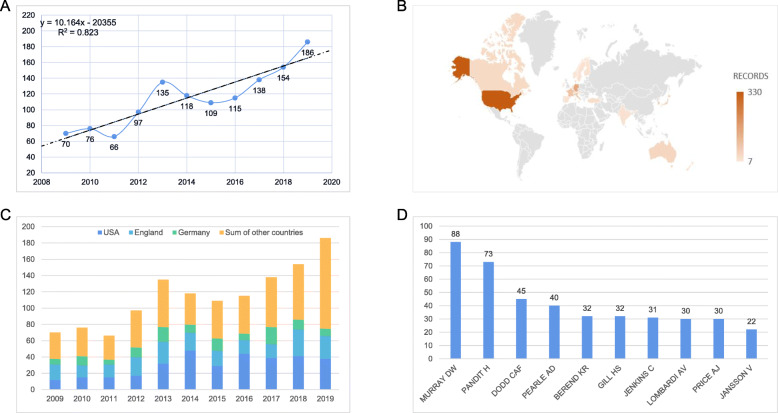
UKA publication: **a**: The model fitting curves of growth trends of UKA publication; **b**:Top 10 countries by volume; **c**: Top 3 countries by volume; **d**: Top 10 authors

A total of 1264 documents were included, and the global volume of publications increased year by year. Among them, the largest number of documents in 2019 (186), accounted for 14.715% of the total. Curve fitting was used to predict the future publication volume according to the time trend, and a time prediction curve model was constructed, with the formula Y = 10.164X-20,355 (Fig. 2a).

#### Countries contributing to global publications

Figure 2b and 2c show that, among the 1264 documents, the United States had the largest number of documents published (330, accounting for 26.108%), followed by the England (251, accounting for 19.858%), and Germany (129, accounting for 10.206%). The total cited frequency (4508) and H-index (34) of papers published in the United States were the highest. The British H-index ranked second, standing at 31, with a total citations of 4041. In Asia, the top three countries that published the most work are South Korea (65), Japan (62), and China (56).

#### Authors contributing to global publications

Figure 2d shows the top 10 authors in this field. The top 3 authors of the papers were British “MURRAY DW”, “PANDIT H” and “DODD CAF”, and they published 88, 73 and 45 articles, respectively [15]. Koh YG (16 papers) of the Yonsei University was the author with the largest volume in Asia [16].

#### Distribution of organizations paying attention to UKA

**Fig. 3:**
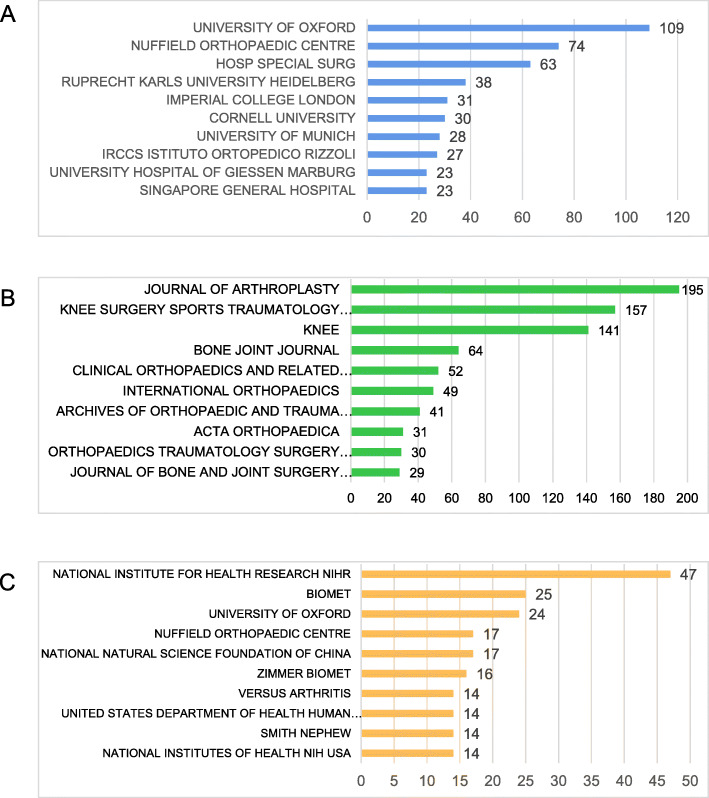
**a**: UKA publication: Top 10 institutions by publication volume; **b**: Top 10 journals by publication volume; **c**: Top 10 funding agencies

Figure 3a shows the top 10 institutions engaging in UKA studies in terms of volume of publications worldwide. Among them, Oxford University has published the most literatures (109 papers), Newfield Orthopaedic Center ranked second (74 papers), and American Special Hospitals ranked third (63 papers) [17]. Singapore General Hospital, Yonsei University, China-Japan Friendship Hospital were the top three institutions in Asia in terms of volume of publications in UKA.

#### Distribution of published journals

In the UKA field, “JOURNAL OF ARTHROPLASTY” magazine has published the most relevant literatures, totaling 195 articles, with an impact factor (IF) of 3.524 in 2018; followed by “KNEE SURGERY SPORTS TRAUMATOLOGY ARTHROSCOPY”, which published 157 relevant papers, and the magazine’s IF was 3.149 in 2018. “KNEE” published 141 related papers, and its IF was 1.762 in 2018. “BONE JOINT JOURNAL” published 64 related papers, with its IF being 4.301 in 2018. “CLINICAL ORTHOPAEDICS AND RELATED RESEARCH” magazine ranked fifth, publishing 52 articles, IF being 4.154. Figure 3b shows the top 10 journals in terms of the number of research literatures on UKA worldwide.

#### Fund support analysis

The National Institutes of Health (47), Bangmei (25), and Oxford University (24) were the top 3 funding agencies of the UKA researches. The National Natural Science Foundation of China (NSFC) ranked 4th, and supported 17 UKA related studies.

### Coupled visual analysis of UKA

#### Country coupling analysis

**Fig. 4:**
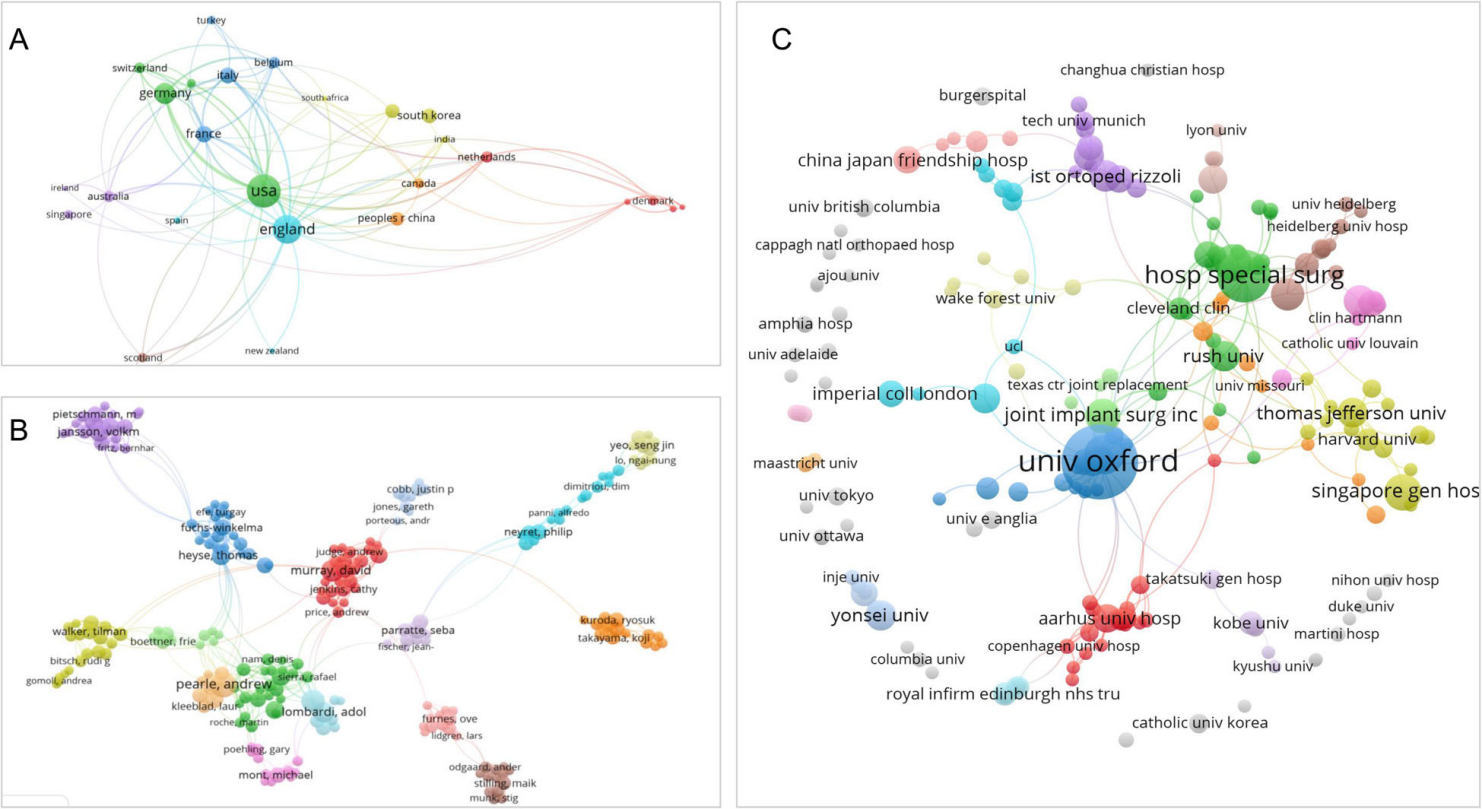
Document coupling analysis. The size of the circle reflects the weight of the literature, and the color of the circle represents the clustering. The more or thicker the lines between the circles, the higher the intensity connection (**a**: country coupling analysis; **b** author coupling analysis; **c**: organization coupling analysis)

33 countries were included by the criterion of at least 3 study records (Fig. 4a). The top five countries in terms of the coupling strength of the literature in this field were the United States, whose total link strength (TLS) was 86, the United Kingdom (72), Germany (58), France (46) and Italy (39).

#### Author coupling analysis

The 1264 articles included involved a total of 4086 authors, and 431 authors were retained against the criterion of being listed at least three times as the author of a document (Fig. 4b). The top three authors in terms of literature coupling strength in this field were MURRAY DW (63 records, TLS 264), DODD CAF (37 records, TLS 180), and PANDIT H (37 records, TLS 163).

#### Organization coupling analysis

All citation materials included covered a total of 1332 institutions, and 208 institutions were included in the analysis according to the criterion of being mentioned at least 3 times in literatures (Fig. 6). The top three institutions in terms of coupling strength in the literature in this field were Oxford University (98 records, TLS 84), American Joint Replacement Center (TLS 39), and Ohio State University (TLS 37).

### Co-occurrence analysis of global UKA keywords

#### Research direction

**Fig. 5:**
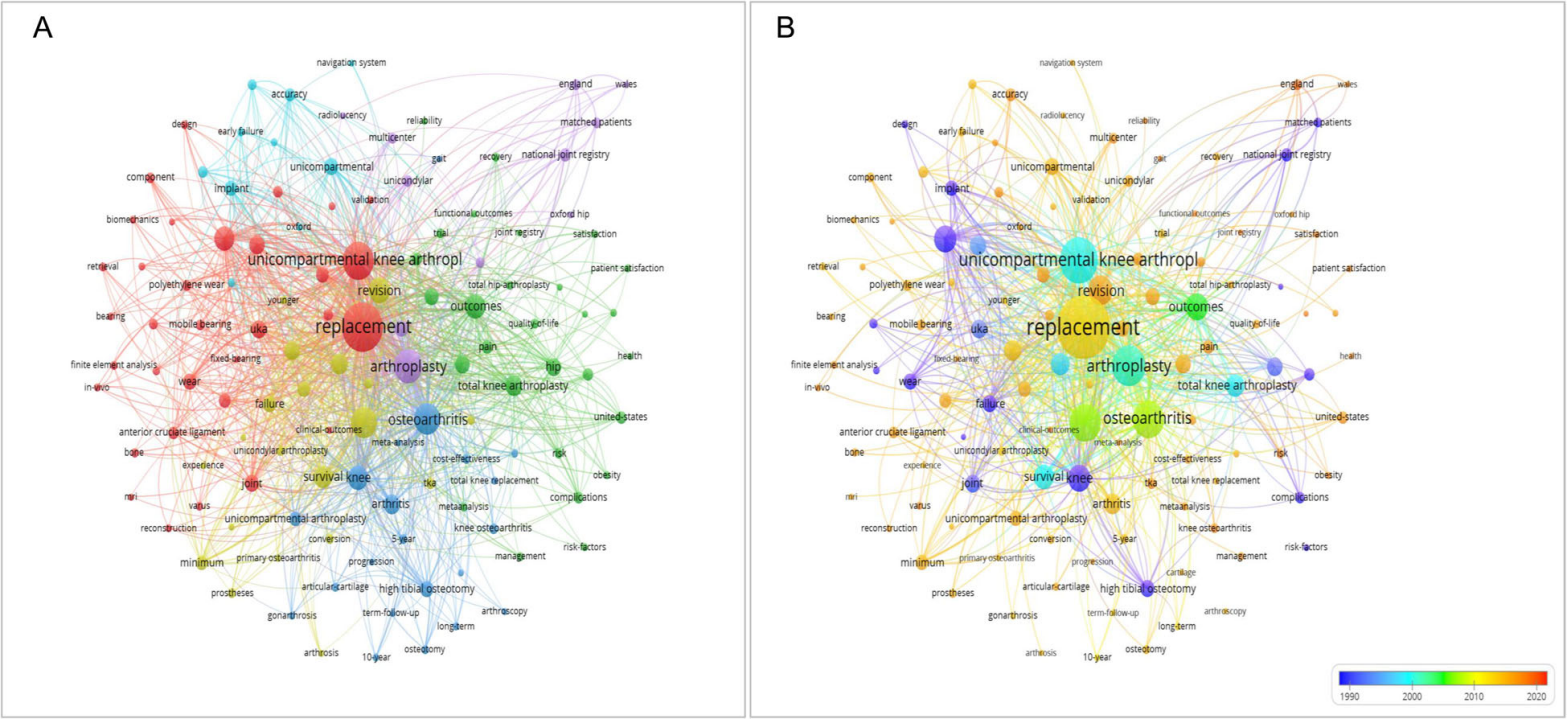
Research direction distribution map

According to the results of keyword clustering, the current research directions of UKA can be divided into six categories: prosthesis design, follow-up investigation, OA etiology, hip-knee association, joint replacement registration, and computer navigation (Fig. 5). In prosthesis design [18] (red circle), the commonly used keywords were “replacement”, “calibration”, “mobile bearing”, “polyethylene wear”. In the follow-up study [19] (yellow circle), the commonly used keywords were “revision”, “survival analysis”, “failure”, “follow-up”, and “minimum”. In the etiology of OA [20] (dark blue circles), the commonly used keywords were “osteoarthritis”, “knee joint”, “cartilage”, and “HTO”. In the hip-knee association study [21, 22] (green circle), “outcomes”, “pain”, “total knee replacement”, and “total hip replacement” were common keywords. The purple circles represent the joint replacement registration clusters [23], and the sky blue circles denote the computer navigation clusters [24, 25].

#### Research hotspots and development trends

**Fig. 6:**
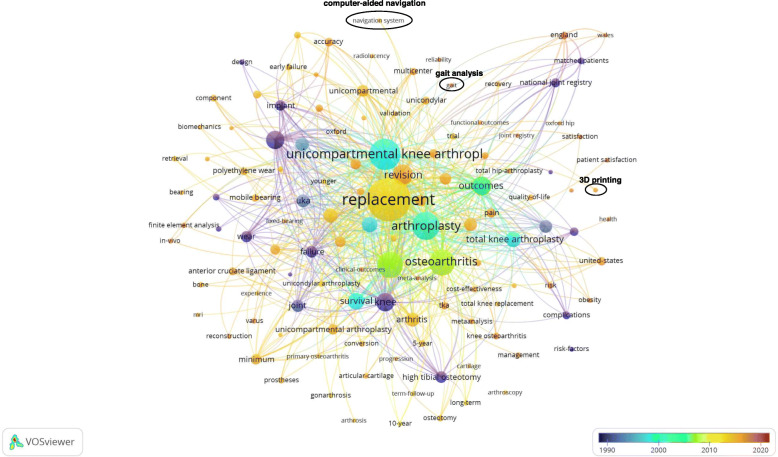
Analysis of research hotspot trends. Over time, the colors in the picture show a purple-green-red change. Keywords in blue belong to early research hotspots, and red parts indicate future research trends.

By dividing different keywords in terms of time, a distribution map of research priorities in different time periods was generated. Figure 6 shows that “computer-aided navigation”, “3D printing”, “gait analysis” are future hotspots in the UKA field.

### Highly-cited papers on UKA

Table 1 lists the top 5 highly-cited papers on UKA published from 2009 to 2019. The first highly-cited paper was published in the *Journal of Bone and Joint Surgery* (*JBJS*) in 2011, and was titled “Revision rates after total joint replacement” [19]. The article reviewed the global joint replacement reports to find out the rate of revision after joint replacement. The average annual revision rate in every 100 patients after hip replacement was 1.29%; the average annual revision rate for the first total knee replacement was 1.26%, and the median revision rate was 1.53%. The revision rate of total hip and knee replacements was virtually the same, with a revision rate being about 6% after five years and 12% after ten years. In 2014, Murray DW published an article in the *Lancet,* titled “Adverse outcomes after total and unicompartmental knee replacement in 101 330 matched patients: a study of data from the National Joint Registry for England and Wales” [26]. The study compared the clinical efficacy in 10,133 patients undergoing TKA or UKA, and the results showed that the 8-year revision rate was higher in UKA group (2.12%) than in TKA group (1.38%). The mortality rate was significantly higher in TKA group than in UKA group at any time point. The complications (including thromboembolism, myocardial infarction, and stroke) and readmission rates were also higher in TKA group than in UKA group. The authors also suggested that surgeons should carefully considered the high revision rate of UKA against TKA in the decision-making of joint replacement, and make effort to lower complications, readmission rates and mortality, and maximize the benefits of UKA in terms of postoperative function.

**Table 1:** Top 5 highly-cited papers on UKA from 2009 to 2019 in the WOS database

Title	Author	Journal	Year	Total cited
Revision rates after total joint replacement	Labek. G	*JBJS*	2011	241
Unicompartmental or total knee replacement	Pydisetty. RV	*JBJS*	2009	172
Minimally invasive Oxford phase 3 unicompartmental knee replacement	Pandit. H	*JBJS*	2011	167
A Second Decade Lifetable Survival Analysis of the Oxford Unicompartmental Knee Arthroplasty	Price. AJ	*Clin Orthop Relat Res*	2011	159
Adverse outcomes after total and unicompartmental knee replacement in 101,330 matched patients: a study of data from the National Joint Registry for England and Wales	Murray. Dw	*Lancet*	2014	156

## Discussion

The “knee-protection” refers to the use of minimally invasive methods to maximize the preservation of the knee joint structure in the treatment of knee joint diseases without destroying the normal mechanical environment and physiological properties of the knee joint [27]. The development of KOA is of a staircase type. Anterior medial osteoarthritis (AMOA) is the most common pathological manifestation of KOA [28], but AMOA is not the best indication for TKA. End-stage KOA should undergo TKA [3, 29]. Therefore, knee protection should be carried out in line with the pathological development of KOA and in phases to achieve maximum patient satisfaction, and treat the knees and forget the knees [30, 31].

Unicompartmental knee arthroplasty and total knee arthroplasty were introduced at the same time in the 1960s. Compared with TKA, UKA has relatively narrow surgical indications and demands higher skills on the part of surgeons [32]. Early UKA, due to immature prosthesis design and surgical methods, the learning curve was relatively long, resulting in a relatively high revision rate and less ideal clinical efficacy [33]. With continuous improvement of UKA prosthesis design, the technology is increasingly standardized, especially, over the past five years, the operation gradually showed its advantages [34].

In the UKA study, the United States and the United Kingdom ranked first and second respectively. More than half of the top 10 authors were from the UK. The University of Oxford published most papers on UKA. The China-Japan Friendship Hospital was the Chinese institution that had the largest number of publications regarding UKA. “JOURNAL OF ARTHROPLASTY” published most papers, with a total of 195 articles appearing in the periodical. “KNEE SURGERY SPORTS TRAUMATOLOGY ARTHROSCOPY”, “KNEE”, “BONE JOINT JOURNAL” were relatively friendly to articles concerning 1to the research direction distribution map, the current research directions of the UKA fall into six categories: prosthesis design, follow-up investigation, OA etiology, hip-knee association, joint replacement registration and computer navigation. In the figure, the circles that stand alone include “arthroscopy” and “gait analysis”, because the newer research direction has yet to be linked to the existing research direction, which also suggests that the new research direction has certain innovative nature, such as UKA in combination with arthroscopy for the treatment of KOA patients and the concepts of knee-protection and sports medicine that are complementary to each other [35]. By superimposing the information of the publication year on the keywords, the research trend is self-evident.

The related series of researches on UKA started as early as 1990, and its research mainly focused on “prosthesis design”, “calibration”, “wear” [36]. After 2010, the UKA gradually became popular, and its main focus was on “follow-up”, “survival analysis”, “risk factors” and other aspects. In 2020, the latest research has shifted from the UKA clinical efficacy analysis to revision surgery, plus hip-knee synergy and patient satisfaction [37]. Research effort is no longer directed to the role of one single factor, but is multifaceted. At the same time, clinical application of high-tech, such as “computer-aided navigation”, “3-D printing”, “gait analysis”,* etc* are future research trends and will become hotspots in the UKA field.

### Deficiencies and outlook

This study had several limitations: First, this study only searched the Web of Science database, and non-English literature was not included in the study, which might result in biases. Second, recently published high-quality documents have low citation frequency due to short after-publication time, and there might exist some errors in the quality assessment of the documents. Finally, bibliometrics only describes the general trend in a certain field. Errors may also occur due to the differences in the statistical algorithms used in different software packages.

While this study might have the aforementioned limitations, we believe that the methodology adopted is of value. Through bibliometrical and visual analysis, research trends and hotspots can be visually displayed, which can serve as guides for the identification of future directions of clinical and basic researches and help improve research competence in the UKA field. “Computer navigation” and “gait analysis” may be the research hotspots for UKA in the future.

## Conclusion

Global trend analysis suggests that UKA research is gradually deepening and the number of papers on the topic is increasing steadily. The USA was the largest contributor to the research of this field. More research effort should be directed at “computer-aided navigation” and “gait analysis”, which may be the popular areas in the research of UKA.

## Supplementary information

**Additional file 1.**

## Data Availability

All data used during the current study are available from the corresponding author on reasonable request.

## Abbreviations

UKA: Unicompartmental Knee Arthroplasty
KOA: Knee Osteoarthritis
WOS: Web of Science
MUR: Medial Unicompartmental Replacement
LUR: Lateral Unicompartmental Replacement
TKA: Total Knee Arthroplasty
H-index: High Citations Index
IF: Impact Factor
NSFC: National Natural Science Foundation of China
TLS: Total Link Strength
JBJS: The Journal of Bone and Joint Surgery
AMOA: Anterior medial osteoarthrit
