# Supplementary material for: Global research status and trends of UKA for knee osteoarthritis: a bibliometric analysis
Source: Arthroplasty. 2020 Jul 24;2:20. doi: 10.1186/s42836-020-00039-3 (PMC8796558; doi:10.1186/s42836-020-00039-3)
Supplement: Supplementary file 1 — Additional file 1. [file 42836_2020_39_MOESM1_ESM.docx]

**The extracted file of supplementary materials**

1. **UKA-FIGURE PPTX**

All figures used in manuscript

1. **Fig1 TIF**

Flow diagram of UKA researches inclusion

1. **Fig2 TIF**

Contributive characteristics on UKA researches

1. **Fig3 TIF** Contributions of organizations , journals, Funding supports to UKA research
2. **Fig4 TIF** Document coupling analysis
3. **Fig5 TIF**

Research direction distribution map

1. **Fig6 TIF**

Analysis of UKA research hotspot trends
